# Editorial: Edible and medicinal plants: From ethnopharmacological practices to interdisciplinary approaches and regulations

**DOI:** 10.3389/fphar.2022.1074511

**Published:** 2022-12-16

**Authors:** Xiaoying Zhang, Alberto C. P. Dias, Norberto Peporine Lopes

**Affiliations:** ^1^ Qinba State Key Laboratory of Biological Resources and Ecological Environment, College of Biological Science and Engineering, Shaanxi University of Technology, Hanzhong, Shaanxi, China; ^2^ Department of Biology, Centre of Molecular and Environmental Biology (CBMA), University of Minho, Campus de Gualtar, Braga, Portugal; ^3^ Department of Biomedical Sciences, Ontario Veterinary College, University of Guelph, Guelph, ON, Canada; ^4^ Núcleo de Pesquisa em Produtos Naturais e Sintéticos (NPPNS), Department of Biomolecular Sciences, Faculty of Pharmaceutical Sciences of Ribeirão Preto, University of São Paulo, RibeirãoPreto, SãoPaulo, Brazil

**Keywords:** edible plants, medicinal plants, pharmacological effect, health benefit, health product, regulation

The investigation on plants that are both medicinal and edible is rooted in different ethno-medicinal systems (Yao et al.) Emerging diseases and health issues, lifestyle and diet changes urge us in seeking novel bioactive substances and formulations from plant sources (Sofowora et al., 2013; Xia et al., 2021). Related study requires interdisciplinary approaches including pharmaceutical, food, medicinal, and plant sciences, and policy study on food and drug administration.

**FIGURE 1:**
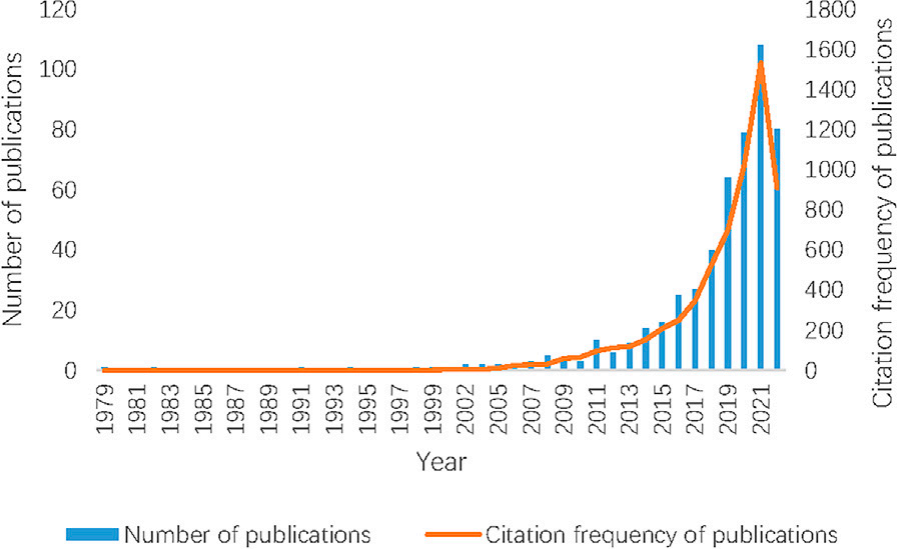
Number and citation frequency of medicine food homology related publications. Note: Data were collected from Web of Science Core Research Topic with the following search strategy: Topical Subject = (“Medicinal and Edible” OR “medicine food homology”) OR Title = (“Medicinal and Edible” OR “medicine food homology”) OR Abstract = (“Medicinal and Edible” OR “medicine food homology”), the language was English. A total of 508 papers were obtained, including 53 reviews and 455 articles. Figure only includes studies that met the inclusion criteria of the search strategy (Data as of Jul. 2022).

Along with the strong increase in the publication of medicine food homology study (Figure 1), research highlights can be observed from the recent publications, which cover the applications of medicine food homology to modern lifestyle related diseases and health concerns, active substances and groups, mushroom study, increasing strengthening on the concept of nutraceuticals, etc. However, there are still a lack of scientific understanding and investigations for active ingredients and their synergistic and networking effects, lack of clear boundary definition in medicinal use and functional use, lack of clear inclusion criteria and regulation policies in the related studies and products. It could be of interest and needs for the scientific community to consider topics such as the development of proper pharmacological and physiological models, the development of novel and practical active substance delivery systems, holistic evaluation of the short term and long-term effects of the plants and the phytoconstituents. Articles merging these topics and ethnopharmacology may be helpful in reaching an international consensus among scientific and industrial communities.

In this Research Topic, we have provided a discussion forum for the community, and received 47 full length article submissions, with 17 papers published, which covers cultivation and pharmacognosy of medicinal and food plants, ethno-pharmacological practices and pharmacological investigation, food function and safety evaluation of selected plants; identification of novel pharmacological and biological effects of plants, strategy on the development of pharmaceutical, nutraceuticals and functional products, reviews as well as general methodology and research advancement (Tables 1, 2).

**TABLE 1:** Edible and medicinal plants and products investigated in the present Research Topic.

Name	Major finding(s)	References
*Crocus sativus* L. (Saffron), Crocetin and its Glycoside Crocin	The anti-angiogenic effects and underlying mechanisms confirmed on human umbilical vein endothelial cells and zebrafish Through VEGFR2/SRC/FAK and VEGFR2/MEK/ERK Signalling Pathways	Zhao etal.
*Maianthemum atropurpureum*, a wild vegetable	The local people’s practice of consuming *Maianthemum atropurpureum* is reasonable due to its high levels of vitamins, minerals, essential amino-acids, and phytochemicals	Xu et al.
Pantao Pill (a traditional Chinese medicines formulation containing Aloes, wood incense, frankincense, myrrh areca, etc.)	Based on Network pharmacology investigations, Pantao Pill can significantly improve the learning and memory abilities of APP/PS1 mice, with mechanisms related to the increase of neurotransmitter acetylcholine and norepinephrine levels, the reduction of the excessive autophagic activation, and the suppression of oxidative stress and excessive apoptotic activity	Xin et al.
*Artemisia capillaris* and 6,7-Dimethylesculetin	*Artemisia capillaris* and 6,7-dimethylesculetin induce Cyp2a5 expression at both mRNA and protein levels. Additionally, 6,7-dimethylesculetin significantly increases cytochrome P450 2a5 expression at the transcriptional level through transactivation by constitutive androstane receptor	Kim et al.
*Morus alba* L. Leaves	Low temperature may be a key trigger in flavonoid biosynthesis of mulberry leaves by increasing the expression of flavonoid biosynthesis-related genes. This study also provided a theoretical basis for the optimal harvest time of mulberry leaves	Xu et al.
*Yeokwisan*, a Standardized Herbal Formula	The study showed the clinical relevance of *Yeokwisan*, in treating functional dyspepsia, especially in promoting gastric emptying but not small intestinal transit. The main mechanisms corresponding to these effects may involve the modulation of the ghrelin pathway and activation of interstitial cells of Cajal in stomach tissue	Hwang et al.
*Sophora davidi* (Franch.) Skeels	*Sophora davidi* (Franch.) Skeels fruits extract demonstrated clear anti–aging effect on d–galactose–induced acute aging in mice, and its mechanism may be relevant to the activation of the SIRT1/p53 signal pathway	Lin et al.
Epimedii Folium	Both species and geographical location variations have impacts on the quality and composition of Epimedii Folium. *Epimedii sagittatum* from Sichuan showed the highest content of bioactive compounds	Li et al.
Edible Bird’s Nest Extract	The extract effectively improved skin wrinkles, is beneficial for skin health and can be used as a skin nutritional supplement	Kim et al.
*Phyllanthus emblica*	Aqueous extract potentially ameliorates non-alcoholic fatty liver disease induced by a choline-deficient, L-amino acid-defined, high-fat diet through a mechanism associated with its modulatory effects on the gut microbiota and microbial metabolism	Luo et al.

**TABLE 2:** Plants or topics reviewed in the present Research Topic

Plant or topic reviewed	Major summary or debate	References
Systematic cross-cultural ethnobotanical knowledge assembly & Genus *Lycium*	A framework for a systematic understanding on any taxon’s ethnobotanical knowledge is proposed. The assembly of the genus *Lycium* indicates the requirement for a documentation-based taxonomic revision to current updated international species checklists	Yao et al.
*Coptis*	Current research situation, knowledge base and research hotspots in *Coptis* research was analysed using Bibliometrics methods	Huang et al.
Emerging Applications of Metabolomics to Assess the Efficacy of Traditional Chinese Medicines for Treating Type 2 Diabetes Mellitus	Metabolomics can be used to systematically explore the pathophysiology of type 2 diabetes mellitus and elucidate overall molecular mechanism underlying the known positive effects of treatment with traditional Chinese medicine. Network pharmacology methods, in combination with experimental pharmacology, can be further used to identify the bioactive ingredients in traditional Chinese medicine and their targets, which could inform the development of new therapies for type 2 diabetes mellitus	Zhang et al.
Tongxinluo Capsule (a Chinese medicinal product composed of *Panax ginseng* C.A.Mey. *Hirudo nipponica* Whitman, *Scolopendra subspinipes mutilans* L. Koch, *Eupolyphaga sinensis* Walker, etc.)	Meta-analysis showed that Tongxinluo could reduce the rate of cardiovascular disease, all-cause mortality and the number and summation of segment depression, decreased serum hypersensitive C-reactive protein level, improve the electrocardiogram abnormalities and clinical efficacy in unstable angina pectoris, relieve the unstable angina pectoris symptoms, as well as increase plasma NO concentrations. Nevertheless, side effects such as gastrointestinal symptoms, bleeding gums, bradycardia, and hypotension still occurred at an inconvenient low frequency	Li et al.
Red Yeast Rice Preparations	Red Yeast Rice Preparations significantly reduce the occurrence of mortality and major adverse cardiovascular events in metabolic syndrome and improve blood glucose, lipid profiles, and blood pressure. Red Yeast Rice Preparations could improve clinical endpoints, and prevent metabolic diseases	Yuan et al.
*Isodon rubescens* (Hemls.) Hara	ethnomedicinal uses, phytochemical composition, pharmacological activity, quality control, and toxicology of *I. rubescens*; updated information for the further development and application as functional food and drug candidate	Chen et al.
Traditional Uses of Animals in the Himalayan Region of Azad Jammu and Kashmir	This study provides baseline data valuable for the conservation of vertebrate and invertebrate diversity in the region of Himalayan of Azad Jammu and Kashmir. It is possible that screening this fauna for medicinally active chemicals could contribute to the development of new animal-based drugs	Faiz et al.

This Research Topic provides a platform and community space for sharing and enlightening the state-of-art discovery and scientific understanding in edible and medicinal plants (Table 1). Efforts are expected for further explorations of this Research Topic in a global perspective with stronger networking. Despite of long-standing application in some cultural systems, the understanding and regulation on edible and medicinal plant are still various and in different approaches among food and drug regulatory authorities of different countries. Consensus on the definition and scope, research and evaluation methodology, and rational application and supervision of edible and medicinal plant are anticipated.
